# Neoadjuvant Chemotherapy Shortens the cfDNA Telomere Length in Breast Cancer Patients

**DOI:** 10.1155/2024/6117394

**Published:** 2024-11-14

**Authors:** İrem Peker Eyüboğlu, Sinan Koca, Betül Çelik, Gökçe Güllü Amuran, M. Ümit Uğurlu, Özkan Alan, Tuğba Akın Telli, Perran Fulden Yumuk, Mustafa Akkiprik

**Affiliations:** ^1^Department of Medical Biology, School of Medicine, Marmara University, Istanbul 34899, Türkiye; ^2^Department of Medical Oncology, Umraniye Education Research Hospital, Istanbul, Türkiye; ^3^Department of General Surgery, School of Medicine, Marmara University, Pendik, Istanbul, Türkiye; ^4^Division of Medical Oncology, Department of Internal Medicine, Koç University School of Medicine, Istanbul, Türkiye; ^5^Division of Medical Oncology, Department of Internal Medicine, Memorial Şişli Hospital, Istanbul, Türkiye

**Keywords:** breast cancer, cfDNA, neoadjuvant chemotherapy, telomere length

## Abstract

**Introduction:** Cancer is a genetic disease that affects people worldwide, and breast cancer is the most common cancer in women. Studies have been conducted on molecular parameters to predict tumor behavior and develop therapeutic strategies. Telomeres, which are at the end of chromosomes, have been studied for their relationship with breast cancer, but more research is needed to understand their role in the disease. Circulating-free DNA (cfDNA) is DNA that is free in the bloodstream and is considered a promising target for early cancer detection, treatment response monitoring, and prognosis assessment. This study is aimed at comparing cfDNA telomere length of breast cancer patients and healthy individuals and analyzing the impact of neoadjuvant chemotherapy on telomere length in cfDNA.

**Materials and Methods:** Blood samples were collected from 33 breast cancer patients undergoing neoadjuvant chemotherapy before and after treatment. The quantitative PCR method is used to measure the average telomere lengths.

**Results:** This study found that the telomere length of cfDNA in breast cancer patients before and after treatment is significantly shorter than in the control group. Neoadjuvant chemotherapy is found to shorten the cfDNA telomere length, especially in the treatment-responsive group.

**Conclusion:** Our study suggests that telomere length in cfDNA may be a useful biomarker for predicting therapy response and possible reoccurrence of the disease in breast cancer patients.

## 1. Introduction

Cancer is a genetic disease that has long been a primary global health concern and continues to affect people worldwide. Breast cancer is a common, highly heterogeneous cancer that originates in the breast tissue, most commonly in the cells of the milk ducts. It can also start in the lobules, the glands that produce milk, or other breast tissue. Breast cancer is the most common cancer in women worldwide, and it can also occur in men, although it is much less common [1, 2]. In recent years, intensive studies have been carried out on molecular parameters that can provide a more precise assessment to predict tumor behavior better and develop therapeutic strategies.

Telomeres were identified in the 1930s as crucial in stabilizing chromosome ends. The telomere is a structured and dynamic complex located at the ends of chromosomes that consists of short, repeating DNA sequences and protective proteins. Telomeres contain tandem GT-rich nucleotide repeat sequences localized to the ends of linear chromosomes [3]. Telomeres prevent the recognition of chromosome ends as damaged DNA that requires repair, thereby maintaining genomic integrity and protecting against chromosomal fusions and rearrangements [4]. The relationship between telomere length and breast cancer susceptibility has been studied, but the results have not been consistently uniform [5]. Every time a cell divides, the telomeres get shorter. When the telomeres get too short, the cell can no longer divide and becomes inactive or dies. This process is thought to play a role in aging and cancer development. Some studies have suggested that telomere length may be associated with an increased risk of certain diseases, such as cancer, but more research is needed to understand the full role of telomeres in health and disease [6, 7]. Evidence suggests that telomeres become dysfunctional during human tumorigenesis, along with telomere shortening in the early stages of cancer. Anaphase bridges, a hallmark of functional telomeres, are low in adenomas but increase significantly in the adenoma–carcinoma transition. In human breast cancer, chromosomal instability rates peak at the ductal carcinoma in situ stage, and anaphase bridges were seen in the preinvasive stages, resulting from chromosomal instability during telomere shortening [8–10].

Circulating-free DNA (cfDNA) is DNA that is found in the bloodstream, and it can come from a variety of sources. The presence of cfDNA in human blood was first described in 1948 and has since been a promising area of research in many medical conditions [11]. The fraction of tumor cfDNA that carries tumor-specific changes is thought to be released into the bloodstream by apoptosis and necrosis, possibly with active secretion during carcinogenesis processes. Apoptotic tumor cells are considered an essential source of cfDNA in the blood [12]. Although most studies calculate telomere length from whole blood DNA, cfDNA is becoming a promising target for early cancer detection, treatment response monitoring, and prognosis assessment. It has been suggested that changes in cfDNA may be more sensitive than peripheral blood leukocyte (PBL) DNA to reflect the cancer process [13].

Some evidence suggests that the telomere length of cfDNA may be associated with breast cancer. Only one study has found that cfDNA from breast cancer patients tends to have shorter telomeres than cfDNA from healthy individuals [14]. It is unclear what the relationship between telomere length and breast cancer might be or whether telomere length could be used as a marker for predicting the risk of breast cancer or for monitoring the progression of the disease. More research is needed to fully understand the role of telomeres in breast cancer and determine the potential usefulness of telomere length as a diagnostic or prognostic marker. In this study, we planned to investigate treatment-altered telomere lengths using cfDNA, which we think helps to understand the tumor profile.

## 2. Materials and Methods

### 2.1. Study Population

Blood samples were collected from 33 female breast cancer patients in Turkey undergoing neoadjuvant chemotherapy. Samples were taken both before treatment and from the same 30 patients 3–4 weeks after the completion of neoadjuvant chemotherapy therapy. Participants were selected based on their diagnosis of breast cancer and their initiation of a new course of neoadjuvant chemotherapy. The average age of the patients was 50. Twenty samples were collected from age- and gender-matched healthy controls. Outcomes were kept anonymous using a random numbering system. All participants in this study have provided written informed consent for their data to be used in this research and for the publication of any results derived from their participation. DNA extraction from plasma was performed with the QIAamp Circulating Nucleic Acid Kit (Qiagen), and DNA was isolated from a 200-*μ*L plasma sample. To minimize bias, investigators were blinded to the clinical status of the participants during sample analysis.

### 2.2. Telomere Length qRT-PCR

Quantitative PCR (Q-PCR) to measure the average telomere length was first used by Cawthon in 2002. In addition to being simple and fast, Q-PCR analysis makes the method advantageous because it requires a small amount of DNA (< 100 ng/sample). The primers we used in our study were taken from the article of Cawthon, the pioneer of this method [15, 16]. Ten nanograms of DNA was used in the Q-PCR reaction performed for telomere and single-copy gene 36B4 using the Light Cycler 480 SYBR Green I Master kit. In 96-well plates, each sample was amplified in a duplicated pattern for two separate genes. Experiments were performed on a real-time PCR device (Light Cycler 480, Roche).

### 2.3. Statistical Analysis

The cfDNA telomere length in patients before the neoadjuvant chemotherapy was compared with the telomere length after the chemotherapy and the healthy controls. Paired and unpaired nonparametric statistical tests were used, and a *p* < 0.05 value was considered significant in statistical analysis, and an evaluation was made at a confidence interval of 0.95.

## 3. Results

**Figure 1:**
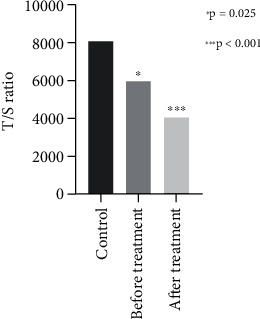
The mean calculated telomere lengths of cfDNAs using the T/S ratio (2^–ΔCt^).

**Figure 2:**
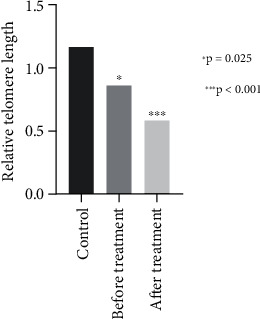
The mean relative telomere lengths calculated by using the 2^–ΔΔCt^ ratio (ΔCt value of the sample to ΔCt value of the control group average).

The current analysis was conducted on 33 women diagnosed with breast cancer with a median age of 51.5.  Table 1 describes the known characteristics of the study population. Among recruited women, we collected 33 plasma samples to examine the telomere length of cfDNA before neoadjuvant chemotherapy and 30 plasma samples after 6 months of neoadjuvant chemotherapy using RT-PCR. The calculated mean telomere length of cfDNAs using the T/S ratio (2^–ΔCt^) is given in Figure 1. The relative telomere length (RTL) is calculated by using 2^–ΔΔCt^ (ΔCt value of sample—ΔCt value of the control group average), and mean values are given in Figure 2 [17].

**Figure 3:**
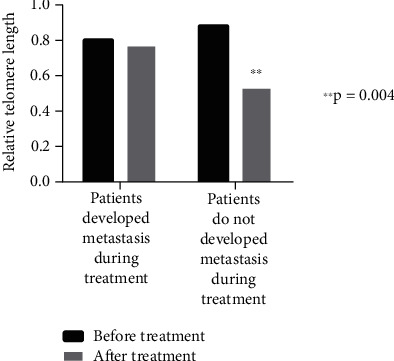
The mean relative telomere length of patients who developed metastasis during treatment and nonmetastatic patients.

The median and mean RTLs of cfDNA in all samples were 0.65 and 0.83, respectively. Before treatment, the median and mean RTLs for breast cancer patients were 0.68 and 0.86, while after treatment, these values dropped to 0.53 and 0.58 (*p* = 0.004 for paired mean RTL). In the control group, the median and mean RTLs were higher at 0.86 and 1.17. The mean RTL in breast cancer patients, both before and after treatment, was significantly lower compared to the control group (*p* = 0.025 and *p* < 0.001, respectively). No significant relationship was found between cfDNA telomere length and the characteristics of the study population. As shown in Figure 3, the telomere lengths of patients who developed metastasis during treatment remained unchanged, whereas the telomere lengths of nonmetastatic patients were significantly shortened after chemotherapy.

## 4. Discussion

This study is aimed at comparing telomere length in cfDNAs of breast cancer patients and healthy individuals and investigating the impact of neoadjuvant chemotherapy on telomere length in cfDNAs. Telomeres are specialized structures found at the ends of chromosomes, and they are essential in maintaining genomic stability and are often used as a marker of cellular aging. Some studies suggest that PBL telomere length may be associated with the development and progression of cancer, while others found a negative or no association between PBL telomere length and cancer at all [18–20]. Cancer cells are generally thought to have longer telomeres than normal cells, possibly due to the telomerase activity in cancer cells that maintains the telomeres and which thus do not shorten as much as normal cells. As a result, cancer cells can divide more than normal cells, leading to uncontrolled growth [21]. cfDNA is DNA that is found in the bloodstream. It can come from a variety of sources, including cells that have died and released their DNA into the bloodstream, as well as cells that are actively shedding DNA. The amount of cfDNA in the bloodstream can vary depending on a person's age, health, and other factors. cfDNA has been studied as a potential tool for cancer diagnosis and monitoring. Some cancer cells release DNA into the bloodstream, and this DNA can be detected in cfDNA [22, 23]. Telomere length in cfDNA may be a useful biomarker for breast cancer because it reflects the overall health and stability of the genome. By measuring telomere length in cfDNA, it may be possible to get a sense of the overall genomic stability of breast cancer cells and how it changes over time. This information could be used to help guide treatment decisions and potentially predict the outcome of treatment. However, more research is needed to fully understand the relationship between telomere length in cfDNA and breast cancer [14].

Our study is aimed at examining the differences in telomere length between cfDNAs in breast cancer patients and healthy controls, as well as investigating how neoadjuvant chemotherapy affects cfDNA telomere lengths. We found out that the telomeric cfDNA levels of the breast cancer patients before and after treatment were significantly lower than the control group statistically (*p* = 0.025 and *p* < 0.001, respectively). A study was conducted in which the RTL in cfDNA of 40 endometrioid endometrial cancer (EC) patients and 31 healthy controls was measured. The results showed that the RTL of EC patients was significantly shorter than that of healthy controls [24]. In a long-term follow-up study of a high-risk population for gastric cancer (GC), it was found that individuals with shorter telomere lengths in their cfDNA had a higher risk of GC progression. Additionally, shorter cfDNA telomere lengths could be detected more than 3 years before a GC diagnosis [25]. Similar to our data, one study found that the level of telomere length of cfDNA was significantly lower in the breast cancer group compared to the healthy group [14].

It is suggested that cancer treatments may contribute to accelerated aging by altering telomere length, but the relationship between cancer treatments and telomere length is not well understood. While some studies revealed PBL telomere shortening on solid tumors after chemotherapy, others found no significant change [26]. Our results clearly show that neoadjuvant chemotherapy shortens the cfDNA telomere length, especially in the treatment-responsive group. It was observed that cfDNA telomere lengths did not change in patients who did not respond to treatment and showed metastasis. There are only six patients whose telomere lengths were found to be longer after treatment. The molecular subgroups of these patients were varied; two of them were Luminal A, two were Luminal B, one of them was Luminal B HER2 enriched, and one of them was triple negative. Four of the patients showed partial response, while two of them showed progression and metastasis during treatment. We could not find any significant association between these data, but if we can study in a large cohort, we believe that we can see a significant correlation.

The exact mechanisms for how chemotherapy may affect telomere length are not fully understood [27]. Paclitaxel causes microtubules to form and become stable, stopping cells in the M phase and causing cell death. Studies have also shown that it causes telomeres to shorten in metastatic melanoma cells. Cell culture results showed telomere erosion in FaDu cells by paclitaxel [28]. Telomere shortening in breast cancer cells after chemotherapy may be indicative of enhanced genomic instability, which could trigger cell death or senescence in highly proliferative cancer cells. This mechanism may underlie the improved response in patients with greater telomere attrition posttreatment. Telomere shortening might result from effective therapy in breast cancer. Further research is necessary to reveal whether chemotherapy-resistant patients' telomere lengths are changing as well as the nonresistant ones. cfDNA telomere length monitoring might be helpful for efficient therapy in cancer patients.

## 5. Conclusion

Our study is aimed at examining the differences in telomere length between cfDNAs in breast cancer patients and healthy controls, as well as studying how neoadjuvant chemotherapy affects cfDNA telomere lengths. We found that the telomeric cfDNA levels of the breast cancer patients before and after treatment were significantly lower than the control group statistically. Our results clearly show that neoadjuvant chemotherapy shortens the cfDNA telomere length, especially in the treatment-responsive group. It was observed that cfDNA telomere lengths did not change in patients who did not respond to treatment and who showed metastasis. There are only six patients whose telomere lengths were found to be longer after treatment. Overall, our study suggests that telomere length in cfDNA may be a useful biomarker primarily for predicting therapy response and possible reoccurrence of the disease. Shortened telomeres after chemotherapy, particularly in patients with a complete response, suggest that telomere length dynamics could reflect treatment efficacy.

## Figures and Tables

**Table 1:** Characteristics of the study population.

**Patient no.**	**Age**	**Lymph node status**	**ER**	**PR**	**HER2**	**Pathologic response**	**Clinical/radiological response**	**Molecular subgroups**	**Neoadjuvant chemotherapy regime**	**Metastasis during treatment**
P1	44	Positive	Positive	Positive	Negative	Partial response	Complete response	Luminal A	4× EC+12× paclitaxel	Negative
P4	45	Positive	Positive	Positive	Positive	Partial response	Partial response	Luminal B	4× EC+12× paclitaxel+Herceptin	Negative
P5	36	Positive	Positive	Negative	Positive	Complete response	Partial response	Luminal B	12× paclitaxel+4× Herceptin+4× EC	Negative
P6						Complete response	Partial response	Luminal B		Negative
P7	56	Positive	Positive	Negative	Negative	Complete response	Complete response	Luminal B	4× EC+12× paclitaxel	Negative
P8	56	Positive	Positive	Negative	Negative	Partial response	Partial response	Luminal B	4× EC+12× paclitaxel	Negative
P9	43	Positive	Positive	Negative	Negative	Complete response	Complete response	Luminal B	4× EC+12× paclitaxel	Negative
P10	48	Negative	Positive	Positive	Negative	Partial response	Partial response	Luminal A	4× EC+12× paclitaxel	Negative
P11	63	Positive	Negative	Negative	Negative	Partial response	Partial response	Triple negative	4× EC+9× paclitaxel	Negative
P12	33	Positive	Positive	Positive	Negative	Partial response	Progressive disease	Luminal A		Positive
P13	80	Negative	Positive	Positive	Negative	Partial response	Partial response	Luminal A	Anastrozole	Negative
P14	39	Positive	Negative	Negative	Positive	Partial response	Progressive disease	HER2 enriched		Negative
P15	39	Positive	Positive	Positive	Positive	Partial response	Complete response	Luminal B	4× EC+12× paclitaxel+4× Herceptin	Negative
P16	53	Positive	Positive	Positive	Positive	Partial response	Partial response	Luminal A	4× EC+12× paclitaxel+4× Herceptin	Negative
P17	61	Positive	Positive	Positive	Negative	Partial response	Partial response	Luminal B	12× paclitaxel+4× EC	Positive
P18	44	Positive	Positive	Negative	Negative	Partial response	Partial response	Luminal B	12× paclitaxel+4× AC	Negative
P19		Positive	Negative	Negative	Negative	Partial response	Progressive disease	Triple negative	4× AC+10× paclitaxel	Positive
P21	34	Negative	Positive	Positive	Negative	Partial response	Partial response	Luminal A	4× EC+12× paclitaxel	Negative
P22	56	Positive	Positive	Negative	Negative	Complete response	Complete response	Luminal B	4× EC+9× paclitaxel	Negative
P23	33	Positive	Negative	Negative	Negative	Complete response	Complete response	Triple negative	4× EC+12× paclitaxel	Negative
P24	61	Positive	Positive	Positive	Negative	Partial response	Partial response	Luminal B	4× EC	Negative
P25	65	Positive	Positive	Negative	Negative	Partial response	Partial response	Luminal B	4× EC+11× paclitaxel	Negative
P26	57	Positive	Negative	Negative	Positive	Complete response	Complete response	HER2 enriched	12× paclitaxel+4× Herceptin+4× AC	Negative
P27	57	Negative	Positive	Negative	Negative	Partial response	Partial response	Luminal B	4× AC	Negative
P28	53	Positive	Negative	Negative	Negative	Partial response	Partial response	Triple negative	4× EC+12× paclitaxel	Negative
P29	38	Positive	Negative	Negative	Positive	Complete response	Complete response	HER2 enriched	12× paclitaxel+6× Herceptin+4× AC	Positive
P30	47	Positive	Positive	Negative	Negative	Partial response	Partial response	Luminal B	12× paclitaxel+4× EC	Positive
P31	56	Positive	Positive	Positive	Positive	Partial response	Partial response	Luminal B	12× paclitaxel+4× Herceptin+4× AC	Negative
P32	64	Positive	Positive	Positive	Negative	Partial response	Progressive disease	Luminal B	4× AC+9× paclitaxel	Negative
P33	61	Positive	Negative	Negative	Positive	Complete response	Partial response	HER2 enriched	10× paclitaxel+7× Herceptin+4× AC	Negative
P34	44	Positive	Negative	Negative	Negative	Partial response	Partial response	Triple negative	4× AC+12× paclitaxel	Positive
P35	50	Negative	Negative	Negative	Positive	Complete response	Complete response	HER2 enriched	4× AC+12× paclitaxel+4× Herceptin	Negative
P36	69	Positive	Positive	Positive	Positive			Luminal B	12× paclitaxel+4× Herceptin+4× AC	Negative

Abbreviations: AC, treatment consists of adriamycin and cyclophosphamide; EC, treatment consists of epirubicin and cyclophosphamide.

## Data Availability

The data that support the findings of this study are available from the corresponding author, İ.P.E., upon request.
